# Statins Lower Lipid Synthesis But Promote Secretion of Cholesterol-Enriched Extracellular Vesicles and Particles

**DOI:** 10.3389/fonc.2022.853063

**Published:** 2022-05-12

**Authors:** Yundi Chen, Yongrui Xu, Jing Wang, Peter Prisinzano, Yuhao Yuan, Fake Lu, Mingfeng Zheng, Wenjun Mao, Yuan Wan

**Affiliations:** ^1^The Pq Laboratory of BiomeDx/Rx, Department of Biomedical Engineering, Binghamton University, Binghamton, NY, United States; ^2^Department of Cardiothoracic Surgery, The Affiliated Wuxi People’s Hospital of Nanjing Medical University, Wuxi, China; ^3^Department of Hematology, Affiliated Drum Tower Hospital of Nanjing University Medical School, Nanjing, China; ^4^Biophotonics and Translational Optical Imaging Lab, Department of Biomedical Engineering, Binghamton University, Binghamton, NY, United States; ^5^Department of Biomedical Engineering, Binghamton University, Binghamton, NY, United States

**Keywords:** extracellular vesicles and particles, statins, cholesterol, lipidomics, ovarian cancer

## Abstract

Lipid droplets are lipid-rich cytosolic organelles that play roles in cell signaling, membrane trafficking, and many other cellular activities. Recent studies revealed that lipid droplets in cancer cells have various biological functions, such as energy production, membrane synthesis, and chemoresistance, thereby fostering cancer progression. Accordingly, the administration of antilipemic agents could improve anti-cancer treatment efficacy given hydrophobic chemotherapeutic drugs could be encapsulated into lipid droplets and then expelled to extracellular space. In this study, we investigated whether statins could promote treatment efficacy of lipid droplet-rich ovarian SKOV-3 cells and the potential influences on generation and composition of cell-derived extracellular vesicles and particles (EVP). Our studies indicate that statins can significantly lower lipid biosynthesis. Moreover, statins can inhibit proliferation, migration, and invasion of SKOV-3 cells and enhance chemosensitivity *in vitro* and *in vivo*. Furthermore, statins can lower EVP secretion but enforce the release of cholesterol-enriched EVPs, which can further lower lipid contents in parental cells. It is the first time that the influence of statins on EVP generation and EVP-lipid composition is observed. Overall, we demonstrated that statins could inhibit lipid production, expel cholesterol to extracellular space *via* EVPs, and improve chemosensitivity.

## Introduction

Lipid droplets (LD) are highly dynamic organelles in almost all kinds of mammalian cells, which play important roles in cell activities (1), including but not limited to energy storage, ATP production, membrane expansion, and signaling (2). The components of LDs are complex, which store thousands of kinds of lipids, lipoproteins, and relevant precursors (3–5). These molecules participate in lipid metabolism and various biological behaviors of cells and tissues. Nevertheless, the biofunctions of these cargos are not fully understood yet. In cancer, metabolism of cancer cells is vigorous as cancer cells have increased energy requirements in comparison to normal cells (6). Moreover, LDs modulate the availability of proteins and signaling lipids, and their dysfunction may lead to disruption of cellular membranes or inappropriate nuclear signaling. Furthermore, LDs function as a place for detoxification. They isolate lipophilic anti-cancer drugs, and thus may contribute to chemoresistance (7–10). Correspondingly, damage or depletion of cytosolic lipid droplets could enhance anti-tumor efficacy through reduction of energy supply, blocking signaling pathways, improving chemosensitivity, and other mechanisms (11). Therefore, it was assumed that LD inhibitors could promote anti-cancer efficacy (10, 12, 13).

Statins are a group of lipid-regulating drugs that can significantly downregulate total cholesterol (TC) (14), low-density lipoprotein (LDL) (15), and triacylglycerol (TG), while upregulating high-density lipoprotein (HDL) (16). Currently, statins are mainly used to treat hyperlipemia and cardiovascular diseases. However, statins have also shown promising anti-tumor efficacy in combination with chemotherapy and immunotherapy (17–21). Although the exact anti-tumor mechanism of statins remains unclear, it might be associated with their lipid-regulation effect. For example, statins are β-hydroxy β-methylglutaryl-CoA (HMG-CoA) inhibitors that can block the mevalonate (MVA) pathway (22). Geranylgeranyl diphosphate, an intermediate product of the MVA pathway, can thereby be down-regulated by statins, which further inhibit the phosphorylation of Ras family proteins (23). The ripple effect may benefit cancer treatment through inhibition of cancer cell proliferation. Furthermore, statins can also inhibit adhesion and invasion of cancer cells through the downregulation of membrane proteins, such as VCAM-1 and integrin-β (24–26). In clinical treatment, the repurposing of well-tolerated and low-toxic statins in combination with chemotherapies have been reported to extend the overall survival of patients with breast cancer (27), ovarian cancer (28), colorectal cancer (29), and other cancers without a resulting increase in cytotoxicity to normal cells. Altogether, combination therapy with statins has been considered as a promising strategy for cancer treatment. It is noteworthy that concern has been expressed regarding the over-prescription of statin drugs as well as the potential for severe adverse effects from statin therapy at high doses (30). The adverse reactions of statins can affect a variety of organs. The most commonly affected ones are musculoskeletal, nerve, skin, gastrointestinal tract, liver, and gallbladder (31). Atorvastatin is a moderately lipid soluble statin with high potent and low toxicity, which can last longer in the body in comparison with other statin drugs (32). For example, atorvastatin cannot cross the blood-brain barrier and may prevent Alzheimer’s disease in the long term without significant adverse effects (32). Therefore, atorvastatin with low dose was investigated in this study.

Small extracellular vesicles and particles (EVP) are lipid-bilayer enclosed particles with a size in the range of 30-300 nm (33). EVPs act as fingerprints of parental cells and they carry proteins, nucleic acids, and lipids (33). They can efficiently deliver these cargos to nearby or distant recipient cells (34). Numerous studies demonstrate that EVPs have a close relationship with tumor development, metastasis, and therapeutic resistance (35). Currently, EVP-derived proteins and nucleic acids are under intense investigation due to potential contributions in cancer liquid biopsy and the molecular mechanisms of cancer. On the other hand, EVPs are also rich with lipid contents, including cholesterol, ceramide, sphingomyelin, and phosphatidylserine (35), which are irreplaceable ingredients in the formation and function of EVPs. For instance, prostate hormone can be delivered to recipient cells *via* EVPs (36), and EVP-derived lipid molecules also participate in intercellular communication (37). Nevertheless, in comparison with proteins and nucleic acids, lipid cargo of EVPs has rarely been investigated thus far. In this study, we investigated the effect of atorvastatin on lipid-enriched ovarian SKOV-3 cells and analyzed lipid contents of EVPs derived from SKOV-3 cells. We found atorvastatin can significantly inhibit SKOV-3 cell proliferation, migration, invasion, and lipid synthesis without obvious cytotoxicity. Moreover, atorvastatin can significantly increase cellular chemosensitivity to paclitaxel (PTX) *in vitro* and *in vivo*. Furthermore, lipidomic sequencing data reveals that atorvastatin can inhibit EVP secretion and enforce the release of cholesterol-enriched EVPs to the extracellular space. It is the first time to observe the influence of statins on EVP generation and lipid composition of EVPs. Our findings reconfirmed statins can enhance anti-cancer treatment efficacy, preliminarily revealed the composition of EVP lipids derived from cancer cells, and may pave a new way for investigating the biologic functions of lipids in cancer biology and drug resistance.

## Results

### Characterization of Cells

**Figure 1:**
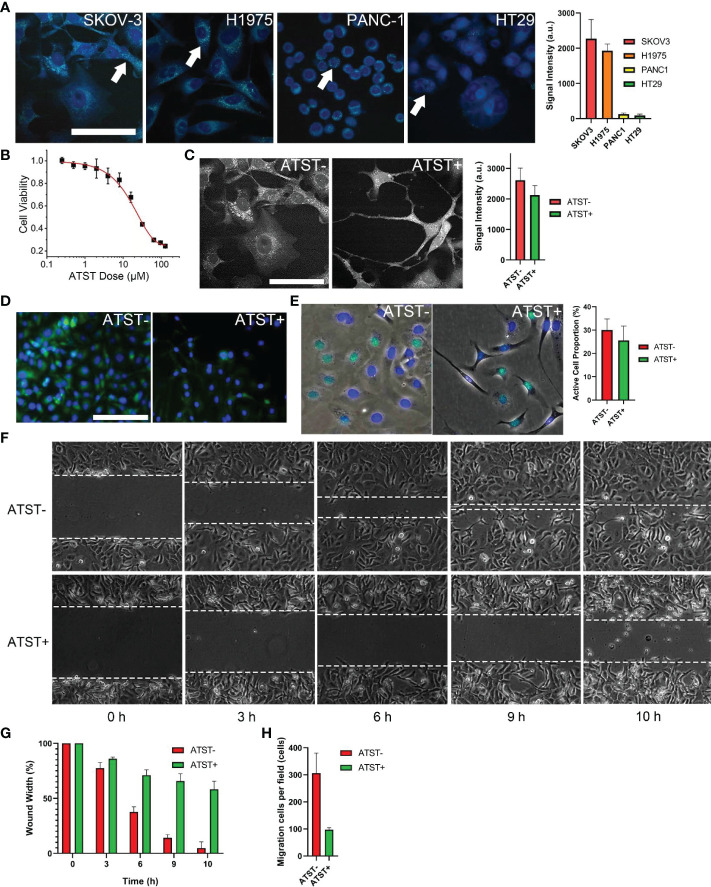
Cell characterization **(A)** Lipid droplets (LD) in four cell lines and quantified signal intensity (scale bar is 10 µm, pseudo color generated by ImageJ). Arrows indicate LDs in cytosol. **(B)** IC_50_ of atorvastatin (ATST) for SKOV-3 cells. **(C)** SKOV-3 LDs under SRS microscopy and quantified signal intensity (scale bar is 10 µm). **(D)** Fluorescence imaging of dye-stained LDs in SKOV-3 cells (scale bar is 100 µm, PKH67: green; DAPI: blue). **(E)** EdU image of SKOV-3 cells (EdU: green; DAPI: blue). **(F)** Representative image of wound healing assay (n=3). **(G)** Quantification of *in vitro* wound healing assay. **(H)** Quantification analysis of trans-well migration assay.

Ovarian cystadenocarcinoma SKOV-3 cell line was reported owning high cytosolic LDs (38). Stimulated Raman scattering (SRS) images also indicated that SKOV-3 cells own the highest LDs in comparison with that of lung adenocarcinoma H1975 cells, colorectal adenocarcinoma HT29 cells, and pancreas ductal adenocarcinoma PANC-1 cells (**Figure 1A**). Therefore, SKOV-3 cells were selected for the following studies. First, we optimized the dose of atorvastatin to avoid direct atorvastatin-induced cytotoxicity. Based on the result of CCK-8 assay, the optimal atorvastatin dose was determined to be 6 μM, at which the viability of SKOV-3 cells was over 90% (**Figure 1B**). After atorvastatin treatment, the morphology of SKOV-3 cells changed from a well-spread shape to a thin and filamentous shape (**Figures 1C, D**). The SRS images of LDs shown the overall signal intensity (average intensity by area) of LDs (bright dots in cytosols) decreased ~16.6% (*p*<0.05) after atorvastatin treatment (**Figure 1C**). The fluorescence images of dye-stained LDs in atorvastatin treated or untreated SKOV-3 cells further confirmed the decrease of LDs in cytosols (**Figure 1D**). Atorvastatin also inhibits SKOV-3 proliferation. EdU cell proliferation assay revealed the average proportion of SKOV-3 cells with active DNA synthesis dropped from 30.1% to 25.6% (*p<*0.05) after treatment with atorvastatin (**Figure 1E**). Wound healing assay demonstrated that atorvastatin could inhibit SKOV-3 cell migration. The average wound gap of untreated cells decreased to 14.1% at the 9-h time point, while the average wound gap of atorvastatin treated SKOV-3 cells remained at 65.7% (*p*<0.01; **Figures 1F, G**). Trans-well assay showed cell trans-well migration decreased ~3.2-fold after atorvastatin treatment (*p*<0.05; **Figure 1H**).

### Combination Therapy *In Vitro* and *In Vivo*

**Figure 2:**
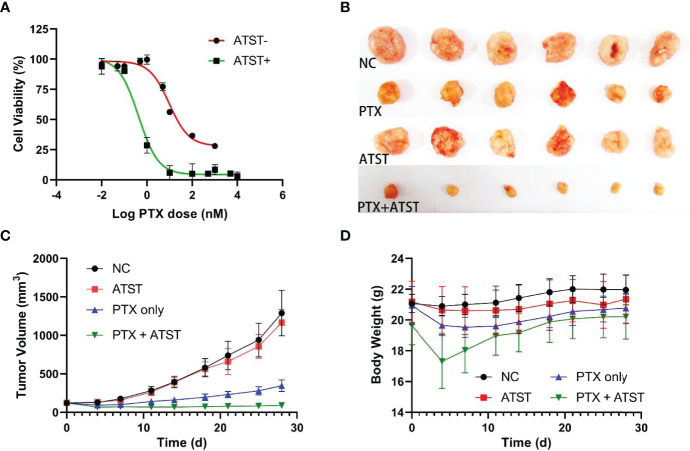
**(A)** IC_50_ of paclitaxel (PTX) in atorvastatin (ATST) treated and untreated SKOV-3 cells, respectively. **(B)** Tumor volume in mice treated with PBS, PTX only, atorvastatin only, and PTX-atorvastatin combination at Day 28. **(C)** Dynamic changes of tumor volume in each group for 28 days. **(D)** Dynamic changes in body weight of mice in 28 days.

SKOV-3 cells in the logarithmic growth phase were used to determine the IC_50_ of PTX. In the atorvastatin treated group, SKOV-3 cells were treated with 6 μM atorvastatin every 12 h for 48 h given the half-life of atorvastatin is ~7 h (39). CCK-8 assay revealed that the IC_50_ of PTX in the statin+ group was 0.43 nM (95% confidence interval: 0.31-0.59 nM), while that of the statin- group was 9.62 nM (95% confidence interval: 7.09-13.21 nM), which suggests the treatment efficacy of PTX in combination with atorvastatin was ~22.4-fold higher than monotherapy with PTX only (**Figure 2A**). Notably, few SKOV-3 cells survived even though PTX dosage was high enough, while almost no cells survived under stress of PTX in combination with atorvastatin, indicating combination therapy could enhance cellular chemosensitivity. Next, the anti-tumor effect of PTX-atorvastatin combination was investigated *in vivo*. On the 28th day, the average tumor volume was 1289.3 mm^3^ in the NC group. In contrast, tumor volume in the PTX only, atorvastatin only, and PTX-atorvastatin groups was 1165.7, 345.8, and 90.0 mm^3^, respectively (**Figures 2B, C**). A significant difference in tumor size was found between the PTX treated groups and the non-PTX treated groups (*p*<0.05). Moreover, a significant difference in tumor size was found between the PTX only group and the PTX-atorvastatin group (*p*<0.05). There was no significant difference in mouse body weight during the 3-week administration period (**Figure 2D**).

### Characterization of EVPs

**Figure 3:**
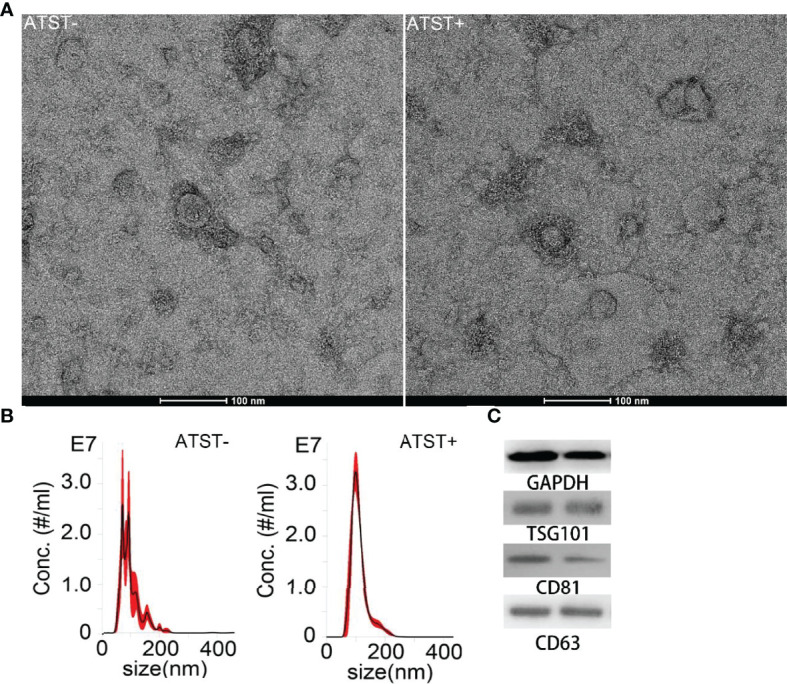
EVP characterization. **(A)** TEM image of EVP derived from cells treated or untreated with atorvastatin (scale bar is 100 nm). **(B)** Size distribution of EVPs derived from cells treated or untreated with atorvastatin. (Left: Atorvastatin- (ATST-) EVPs; Right: ATST+ EVPs). **(C)** Western blotting analysis of EVPs derived from cells treated (right) or untreated (left) with atorvastatin.

The SKOV-3 derived EVPs were characterized by TEM after isolation and purification. EVPs showed a typical saucer shape under microscope (**Figure 3A**). EVP size ranged from 30 nm to ~300 nm, measured by nanoparticle tracking analyses. The average size of EVPs in the control group and EVPs in the atorvastatin treated group were 109.9 nm and 102.1 nm (*p*<0.001), respectively (**Figure 3B**). Moreover, atorvastatin decreased EVP generation rate to 1.53×10^4^ EVP/cell/h compared to 3.29×10^4^ EVP/cell/h in the control group (*p*<0.001). The internal reference protein, GAPDH, as well as classical EVP protein markers, including TSG101, CD81, and CD63 extracted from EVP protein lysates and cell lysates were detected by Western blot (**Figure 3C**). The expression level of these proteins did not show significant alterations (<1.3-fold; *p*>0.05).

### Lipidomic Sequencing

**Figure 4:**
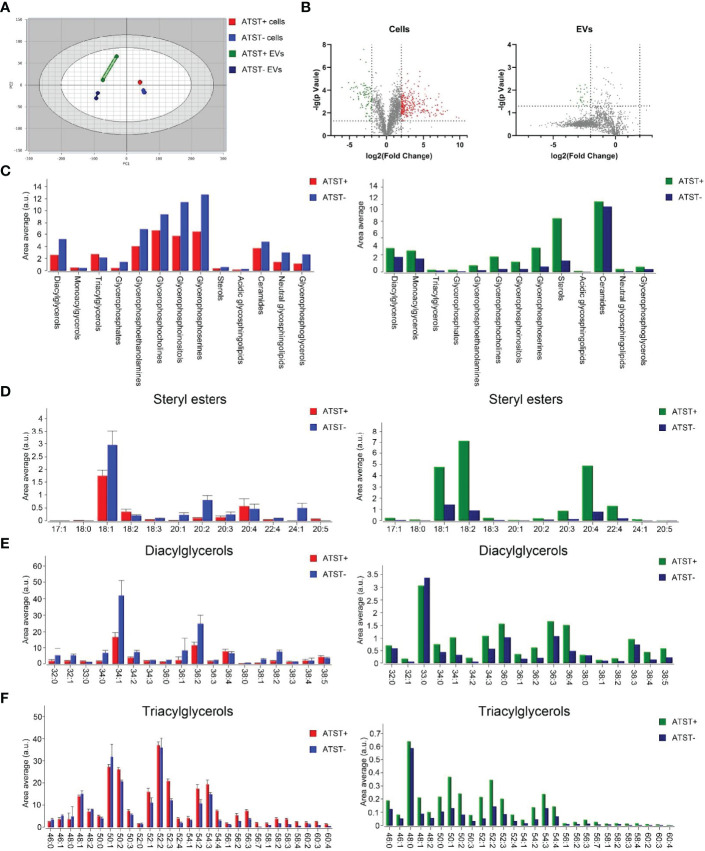
**(A)** PCA plot lipid feature of each group (Red: Atorvastatin+ (ATST+) cells; Blue: ATST- cells; Green: ATST+ EVPs; Blue: ATST- EVPs). **(B)** Volcano plot comparing the lipid composition in cell samples (left) and EVP samples (right). **(C)** Lipid abundance plot by lipids in cell samples and EVP samples. **(D)** Difference in abundances of stearyl ester between cell samples (left) and EVP samples (right). **(E)** Difference in abundances of DG between cell samples (left) and EVP samples (right). **(F)** Difference in abundances of TG between cell samples (left) and EVP samples (right).

Lipidomic sequencing was used to analyze SKOV-3 cells derived lipids (statin-), atorvastatin treated SKOV-3 cells derived lipids (statin+), SKOV-3 EVPs derived lipids (EVP-), and atorvastatin treated SKOV-3 EVPs derived lipid (EVP+). A total of 2608 different lipids were identified from cells, and 2124 lipids were identified from EVPs based on untargeted lipidomic analysis. The differences in lipid profiles between four groups was visualized by principal component analysis (PCA) which revealed significant intergroup difference (**Figure 4A**). The intra‐group variation in lipids derived from cells was lower than that derived from EVPs (**Figure 4A**). The highest batch-to-batch variation in lipid contents was observed in the EVP+ group. In two cell groups (statin+ vs. statin-), volcano plot shows 891 downregulated lipids and 275 upregulated lipids after statins treatment (fold change >2). In two EVP groups (EVP+ vs. EVP-), there were 1430 upregulated lipids and 15 downregulated lipids after statins treatment (fold change >2) (**Figure 4B**). The result of lipid abundance analysis showed the expression level of common high abundance lipids, such as sterol, diacylglycerols (DG), ceramide, and phosphorylated esters, significantly decreased after atorvastatin treatment in SKOV-3 cells, but significantly increased in EVPs (**Figure 4C**). Moreover, in terms of sterol, cholesteryl easter (CE) 18:1 and CE 18:2 showed significant differences both in cells and EVPs (**Figure 4D**). TG was slightly increased, while DG was significantly decreased in cells (**Figures 4E, F**).

## Discussion

Lipid metabolism is closely involved in cellular functions (40). However, the relationship between lipid metabolism and tumors is overly complex with very limited understanding of relevant mechanisms thus far. Undeniably, altered lipid metabolism is among the most prominent metabolic alterations in cancer. Enhanced uptake or synthesis of lipids contributes to rapid cancer cell growth, tumor formation, and drug resistance. It has been reported that many tumor cells, including ovarian cancer, showed increased cholesterols uptake and synthesis compared with normal cells (41). Naturally, LDs were accumulated in cytosol of tumor cells as energy source under stress. On the other hand, cholesterol metabolism depends on the MVA pathway which is heavily involved in the synthesis of various cellular membrane components and organelles. In tumor cells, the MVA pathway loses its feedback inhibition, and thus a large amount of cholesterol is synthesized. The synthesized cholesterol is further used for membrane formation, which supports fast division of tumor cells. Moreover, cholesterol is enriched in lipid raft (42). The massive production of cholesterol thereby facilitates the expression of several tumor related signaling proteins, such as CD24 (promoting angiogenesis) (43), TGFβ (promoting epithelial-mesenchymal transition) (44), matrix metallopeptidase (promoting migration) (45), and CD44 (promoting adhesion) (46), which need to anchor on a lipid raft. In addition, studies found that lipids reduce chemosensitivity of tumor cells. For instance, tyrosine kinase inhibitor (TKI) resistant lung cancer cell lines, HCC827GR, H1975, and PC9GR, have more LDs than TKI sensitive cell lines, and cancer cells treated with oleic acid can restore TKI sensitivity (47). Overall, cancer cells demand lipids for proliferation, migration, invasion, and drug resistance. Correspondingly, we hypothesized that reducing or altering lipid metabolism could inhibit cancer development and restore drug sensitivity.

Repurposing of statins for cancer treatment may achieve the above-mentioned goals. First, statins can inhibit HMG CoA reductase, further restrain MVA pathway and lipid synthesis, and finally reduce cellular activities (48). Second, the expression of certain proteins, e.g., hormone receptors, can be downregulated by statins (49, 50), and thus affect cancer cells. Third, at the nucleic acid level statins can induce the expression of certain small RNAs that can down-regulate the expression of LDL receptors and thus inhibit cancer cells *via* reduced cholesterol intake (51). Fourth, statins may also lower ATP production and inhibit efflux pumps on membranes. Efflux pumps require ATP to transport foreigners, including chemical drugs, from cytosol to the extracellular space (52). The downregulation of intracellular cholesterol level induced by statins can activate sterol regulatory element-binding protein-2 (SEBP2) gene which can further promote lipoprotein uptake. Meanwhile, the SEBP2 can downregulate the expression of efflux pumps (53), and thus statins can retain chemotherapy drugs within the cytosol. Last, statins can reduce the number of cytosolic LDs. Instead of being trapped in LDs and expelled to extracellular space through exocytosis, lipophilic anti-cancer drugs can efficiently stay within cytosols, interact with target molecules, and exert therapeutic functions. In this study, we observed that atorvastatin can significantly inhibit SKOV-3 cells and increase the chemosensitivity *in vitro* and *in vivo*, which is in line with previous studies (54–57). Notably, we optimized atorvastatin dose and ensured atorvastatin did not show significant cytotoxicity. Therefore, the improved cytotoxicity of PTX was not contributed by the toxicity of atorvastatin itself. We speculated the aforementioned statins’ functions might be exerted simultaneously in assisted chemotherapy, although the exact mechanisms are unclear.

We noticed the EVP generation rate was significantly reduced by atorvastatin. Given EVP cargos derived from cancer cells can promote cancer metastasis, this finding indicates atorvastatin may inhibit the formation of premetastatic niche, influence tumor microenvironment, and decrease organotropic metastasis. As to the decreased EVP generation rate, it might be caused by altered cholesterol level in SKOV-3 cells. Cholesterol is an essential component of mammalian cells. The concentration of cholesterol on plasma membranes is much higher than that in other cellular compartments (58). Because atorvastatin directly inhibits synthesis and uptake of cholesterol, inherently membrane synthesis can be inhibited. Subsequently, EVP production is affected due to inadequate membranes for EVP assembly and release. Moreover, atorvastatin participates in G protein modification, which negatively influences the self-assembly of cytoskeletal components and the transportation of lipoproteins (59). Consequently, the EVP generation can be decreased in a non-lipid dependent way.

Lipid analysis of EVP reported that EVP-derived lipid contents are different from that of parental cells (60), which may relate to the biogenesis of EVPs (61). Therefore, we further performed lipid sequencing. Overall, lipid sequencing data validated the atorvastatin lowered the lipid abundance in cells. But atorvastatin did not always downregulate lipids. For example, the abundance of DG decreased while that of TG raised (**Figure 3C**). Although both TG and DG are the key components of LDs, they play different roles in cellular functions. TG often functions as energy storage and is more related to maintaining cell survival. When the cell is under stress, TG will be upregulated to maintain a relatively mild metabolic environment, which was exactly in line with our lipid sequencing data. Moreover, TG in LD can be hydrolyzed to form DG. In contrast, phosphorylated DG can form a series of new second messengers and participate in various signaling pathways. DG itself can also bind to a variety of receptors which are mainly related to cell proliferation. In living cells, DG and TG regulate each other, and thus cells can keep a balance between growth and proliferation. Under the pressure of atorvastatin, the imbalance of the ratio of TG and DG can significantly alter the cell cycle of SKOV-3 cells. The altered cellular cycle may convert a cold tumor to a hot tumor, which can increase sensitivity to immunotherapy to a certain extent.

On the contrary, the abundance of lipids was significantly increased in EVPs derived from atorvastatin-treated SKOV-3 cells (**Figure 3C**). It was reported that cholesterol in excess of the current cellular demand is either exported from the cell by ATP-binding cassette transporters, or converted to less toxic cholesteryl esters and then stored in lipid droplets or secreted within lipoproteins (62). Therefore, we assume surplus lipid components, especially stearyl esters, can attach onto lipoproteins, and the complex can be further encapsulated into EVPs for extracellular secretion. Moreover, the large number of lipid rafts composed by sphingolipids and cholesterol may promote lipid raft-mediated EVP endocytosis of recipient cells (63). Given macrophages are primarily responsible for the rapid clearance of EVPs from the bloodstream, which drastically limits the amount of EVPs that are available to reach the recipient cells and tissues (64), we speculate that atorvastatin-induced lipid-enriched EVPs could be efficiently cleared by macrophages (65). The risk of cancer metastasis thereby can be further reduced. On the other hand, because many receptors are anchored on the lipid raft, we assume that it would be more feasible to screen tumor biomarkers by analyzing atorvastatin-induced lipid-enriched EVPs derived from cancer cells. The results of untargeted lipid sequencing also confirmed that parental cells and EVPs have a significantly different lipid composition. The abundance of lipids other than cholesterol esters in cells was significantly higher than that in EVPs, suggesting that EVPs are rich with lipid rafts but poor with energy storage.

In conclusion, atorvastatin can significantly inhibit ovarian SKOV-3 cell proliferation, migration, and invasion. Meanwhile atorvastatin increases chemosensitivity of SKOV-3 cell *in vitro* and *in vivo*. Moreover, atorvastatin can reduce EVP generation, which may lower the risk of cancer metastasis. Lipid sequencing data revealed the significant differences in lipids derived from parental cells and respective EVPs. Only TG level was upregulated after atorvastatin treatment. In contrast, all lipids were upregulated in EVPs derived from atorvastatin treated SKOV-3 cells. The potential influence of these changes is unclear yet, but we speculated these ripple effects may benefit atorvastatin treated patients from chemotherapy and immunotherapy. In our future work, we will further explore the association between EVP-derived lipids and cancer progression, screen potential EVP lipids as diagnosis or prognosis markers, and investigate the treatment efficacy of immunotherapy in combination with statins.

## Methods and Materials

### Cell Culture

The human cancer cell lines, including SKOV-3 cells, H1975 cells, HT29 cells, and PANC-1 cells, passed the mycoplasma test throughout the whole experiments. Cells were cultured in DMEM with 5% FBS and 100 IU/mL penicillin-streptomycin at 37°C, 5% CO_2_, and 95% humidity. Before collecting EVPs, the cells were kept in FBS-free medium for at least 24 h.

### Optimization of Atorvastatin Dose

Approximately 1000 SKOV-3 cells were seeded into each single well in a 96 well plate. The plate was incubated at 37°C, 5% CO_2_, and 95% humidity for 24 h. Then, 100 μl of atorvastatin with concentration gradient was added to each well. After incubation for 48 h, 10 μl of CCK8 solution was add to each well followed by 1 h incubation at 37°C. The absorbance at 450 nm was read to calculate cell viability. Three biological replicates were prepared.

### Lipid Droplet Imaging

The cells were divided into two groups. In the experimental group, cells were treated with 6 µM atorvastatin for 48 h followed by fixation with 4% paraformaldehyde. Images were taken by homemade SRS microscopy which was built by Biophotonics and Translational Optical Imaging Lab. Signal intensities of LDs were analyzed with ImageJ.

### Cell Proliferation Assay

The protocol of EdU can be found elsewhere. In short, an appropriate number of cells at logarithmic phase were seeded in two 60-mm petri dishes. In the experimental group, 6 μM of statin was supplied while normal DMEM was supplied in the control group. After 48 h incubation, prewarmed 37°C EdU working solution was added. The dishes were continually incubated for 2 h before fixing by 4% paraformaldehyde. Then, the dishes were washed by PBS 3 times followed by treating with 0.3% Triton X-100 solved in PBS. Triton X-100 was removed, and the dishes were washed another 3 times by PBS. Before imaging, click reaction buffer, CuSO_4_, biotin azide, and click additive solution were added to each dish based on the manual. The cells were further stained with DAPI. The image was analyzed by ImageJ.

### Cell Wound Healing Assay

Approximately 5×10^5^ cells were seeded in a 6 well plate and incubated overnight for firm surface attachment. The wound was created by scratching with a tip. The plate was washed 3 times with PBS to remove detached cells and debris. Images were taken at 0-, 3-, 6-, 9-, and 10-time points under microscope. The data were analyzed by MATLAB.

### EVP Harvest and Characterization

In total, 200 ml of cell culture supernatant was collected followed by centrifugation to remove intact cells (500 g for 5 min) and cellular debris (20,000 g for 15 min). EVPs were isolated by ultracentrifugation following MISEV2018 EVP isolation protocol. The EVP pellets were resuspended in 30 µl of PBS and cryopreserved at -80°C. EVP concentration and size distribution were determined with Nanosight NS300. Five microliters of EVP samples was placed on 300 mesh grids and incubated for 3 min at room temperature (RT). Excess samples were blotted with filter paper and stained with 1% uranyl acetate for 5 min. Samples were then examined under TEM (Hitachi). The Western blot was routinely performed. After lysis with RIPA buffer, Mini-PROTEAN Tetra Handcast System (BioRad) and Trans-Blot Turbo Transfer System (BioRad) were used for electrophoresis and subsequent transferring. The protein blot was blocked for 1 h with 5% skimmed milk in PBS/0.05% Tween 20 and incubated for 6 h at 4°C with Santa Cruz Biotechnology HRP conjugated antibodies against TSG-101 (sc-7964, 1:500), CD81 (sc-166029,1:500), CD63 (sc-100304, 1:500), and GAPDH (sc-47724, 1:1000). Samples were washed with PBS/0.05% Tween for 10 min 3 times. Blots were developed with chemiluminescence (BioRad).

### Lipid Extraction

Two milliliters of mixture containing chloroform, methanol, and water (2:1:1) was added to sedimented cells followed by vortex and centrifugation (20,000 g for 10 min). The lower organic phase was collected. Approximately 50 μL of formic acid and 1 mL of chloroform-methanol-water mixture were added to the remaining aqueous phase solution followed by vortex and centrifugation (20,000 g for 10 min). The organic phases obtained twice were mixed and dried. EVP derived lipids were extracted following the same protocol. Lipid sequencing was performed by Cayman Chemical Company.

### *In Vitro* Therapy

All animal experiments were approved by and performed in accordance with guidelines from the Institutional Animal Care and Use Committee (IACUC) of the Model Animal Research Center of the Wuxi People’s Hospital Affiliated with Nanjing Medical University (Wuxi, China). The approval number was 2021-0168. The BALb/c nude mice age in 6-8 weeks with weights of 18-22 g were used. To establish a xenograft model, adult female BALb/c nude mice were s.c. injected under anesthesia with 5×10^6^ SKOV-3 cells resuspended in mixture of Matrigel (Corning) and PBS (1:1). The mice were divided into 4 groups and were respectively injected with PBS (negative control, NC group), atorvastatin (ATST), PTX (PTX group), and a mixture of PTX and atorvastatin (PTX+ATST group). The body weight and tumor size of each group were measured every 84 h.

### Statistics

Data analyses were carried out using SPSS 23 software program. The statistical significance was determined by Student’s t-test and ANOVA test. All tests were two-sided, and *p*-values <0.05 were considered statistically significant.

## Data Availability Statement

The original contributions presented in the study are included in the article/supplementary material. Further inquiries can be directed to the corresponding authors.

## Author Contributions

YC, WM, and YW designed the research. YC and YX conducted experiments and analyzed data. JW, PP, YY FL and MZ assisted in experiments. YC, YX, and YW wrote the manuscript. All authors contributed to the article and approved the submitted version.

## Funding

The work was partially supported by the National Cancer Institute 1R01CA230339 and 1R37CA255948 and the Natural Science Foundation of Jiangsu Province (BK20212012 and BK20210068) and the Top Talent Support Program for Young and Middle-Aged People of Wuxi Health Committee (HB2020003).

## Conflict of Interest

The authors declare that the research was conducted in the absence of any commercial or financial relationships that could be construed as a potential conflict of interest.

## Publisher’s Note

All claims expressed in this article are solely those of the authors and do not necessarily represent those of their affiliated organizations, or those of the publisher, the editors and the reviewers. Any product that may be evaluated in this article, or claim that may be made by its manufacturer, is not guaranteed or endorsed by the publisher.
